# A conditionally replicating SIV variant that can be used to study the mechanism of protection conferred by live attenuated SIV vaccines

**DOI:** 10.1186/1742-4690-8-S2-P16

**Published:** 2011-10-03

**Authors:** Atze T Das, Neil Berry, Bep Klaver, Mark Page, Richard Stebbings, Debbie Ferguson, Martin P Cranage, Neil Almond, Ben Berkhout

**Affiliations:** 1 AMC- University of Amsterdam, Laboratory of Experimental Virology, Amsterdam, 1105AZ, Netherlands; 2 HPA- NIBSC, Division of Retrovirology, Potters Bar, Herts, EN6 3QG, UK; 3 St George's, University of London, Centre for Infection and Immunity London, SW17 ORE, UK

## Background

Live Attenuated SIV confers potent protection against wild-type SIV challenge. However the mechanism of protection is poorly understood and does not appear to correlate with adaptive immune responses. We have constructed a novel conditionally live SIVmac239 variant that replicates exclusively when doxycycline(dox) is administered [1,2]. This SIV-rtTA variant is used to investigate the role of duration and persistence of vaccination in protection against homologous and heterologous wild-type virus challenge.

## Materials and methods

Mauritian cynomolgus macaques were vaccinated with SIV-rtTA and subsequently challenged with the heterologous wild-type virus SIVsmE660. Indian rhesus macaques were vaccinated with SIV-rtTA and subsequently challenged with wild-type SIVmac239.

## Results and conclusions

In naive cynomolgus macaques, SIVsmE660 establishes high peak and steady-state viraemia. After 20 weeks vaccination with SIV-rtTA in the presence of dox, 6/6 animals were protected against SIVsmE660. By contrast only 1/6 animals were protected after 3 weeks vaccination in the presence of dox. The role of persisting vaccine virus replication was investigated by vaccinating for 3 weeks with SIV-rtTA in the presence of dox, removing doxfor 17 weeks and subsequent challenging with SIVsmE660. In this group, 3/6 vaccinates were protected against detectable infection with SIVsmE660 in the periphery. These data demonstrate that live attenuated SIV can protect against a vigorously replicating, heterologous challenge virus (SIVsmE660) and that a longer duration of vaccination is beneficial, even when the vaccine-virus is apparently 'switched-off'.

Vaccination of rhesus macaques with the same SIV-rtTA vaccine for 6 months in the presence of dox protected only 2/8 vaccinates against homologous challenge with wild-type SIVmac239. Remarkably, both protected animals demonstrated more persistent viral load kinetics upon vaccination, which confirms that prolonged replication of the vaccine virus improves protection. Sequence recovery of SIV-rtTA indicated an absence of mutations in the rtTA gene that would impact on doxycycline dependency. The presence of only minimal changes elsewhere indicated that SIV-rtTA was genetically stable in vivo. Since the genetics and antigenic relatedness of vaccine and challenge virus does not appear to predict the outcome of these vaccine studies, the kinetics and relative persistence of vaccine virus replication and the role of innate anti-retroviral responses are being evaluated.
